# Acknowledgment to Reviewers of *Tropical Medicine and Infectious Disease* in 2021

**DOI:** 10.3390/tropicalmed7020017

**Published:** 2022-01-27

**Authors:** TropicalMed Editorial Office

**Affiliations:** MDPI AG, St. Alban-Anlage 66, 4052 Basel, Switzerland

Rigorous peer-reviews are the basis of high-quality academic publishing. Thanks to the great efforts of our reviewers, *TropicalMed* was able to maintain its standards for the high quality of its published papers. Thanks to the contribution of our reviewers, in 2021, the median time to first decision was 17 days and the median time to publication was 42 days. The editors would like to extend their gratitude and recognition to the following reviewers for their precious time and dedication, regardless of whether the papers they reviewed were finally published:
Abhilasha KarkeyKrishna Kishore UmapathiAbia Akebe Luther KingKristine GebbieAdela Enache-AngoulvantKrzysztof OrczykAdina Turcu-StiolicaKuldeep GuptaAhmad MouradKun-Hsien TsaiÁkos JerzseleKurt G.M. De CramerAlberto Arnedo-PenaLaura E. ViaAldemir B. Oliveira-FilhoLaurence Toutous TrelluAleksandra Burkowska-ButLavinia Schuler FacciniAlessandro FeolaLeandro Ferracini CabralAlice MichieLeon HugoAmar DebboucheLeona GilbertAmina KhatunLeonardo PaganiAmy GilbertLiam ReynoldsAna Cecília Ribeiro CruzLingpeng ShanAna PerisinLingxiao XieAnders C. BoydLisiane Morelia Weide AcostaAndré P. WolffLizette KoekemoerAndrea CassoniLorna LealAndrea CioffiLuc ParisAndrea CoppolaLuca BusettoAndrea RossaneseLuca CoppetaAndreas NeumayrLuca VillaAndrew PetersLucie PaloqueAndrew PikeLuigi MeccarielloAndroula PavliLuis ChavesAndrzej TukiendorfMagnus GrabeAndy AlhassanMahmoud KandeelAngela BarbosaManuela SilvaAnisuzzaman AnisuzzamanMāra PilmaneAnnetta ZintlMarcello TagliaviaAnnette JurkeMarcelo Andreetta CorralAnthony GuihurMarcelo Palma SirciliAntonia EfstathiouMarcos Bryan HeinemannAntonio BaldassarreMarcus ScottiAntonio Carlos PaesMaria Alfonsa CavaleraAntonio Gregorio Dias JuniorMaría J. García-MurriaAntonio ZuritaMaria Teresa Armúa-FernándezApiporn Thinkhamrop SuwannatraiMaria Teresa ManfrediArmanda BastosMarialaura CorrenteAssaf RokneyMarie VarloudAttila D. SándorMarino VilovićAugustin Kadima EbejaMarios PapadakisAyman KhattabMarisol OcampoAyman MahmoudMarius MasiulisBaicus AndaMartin TaylorBayodé Roméo AdégbitèMasaki OtaBela KocsisMateusz M. PlucinskiBenjamin CullMathieu NacherBethany Hedt-GauthierMatteo BolcatoBettina WollankeMatteo RiccòBo NiklassonMatthew GrantBongju KimMax BachmannBronislava VichovaMax Carlos Ramírez SotoCarla Bianca Luena VictorioMelissa Agsalda-GarciaCarlo GenoveseMerete MarkvartCarmen de KockMichael PadgetCarmen SolcanMichał Kazimierz ChojnickiCarol Yen Chin LinMichel LedizetCarolina DaviesMichele FioreCassiano Felippe Gonçalves-de-AlbuquerqueMichele LunardiCatrin MooreMiklós BitayChee Keng MokMilan KolářCheng-Chieh YenMohammad Abdul Motalib MominChristian AuerMohammed GrawishChristian NsanzabanaMohammed InayathullahClaudia MarottaMonia CocchiConxita MestresMonika Pogány SimonováDaniela BoccoliniMoosa PatelDanilo Oliveira CarvalhoMuhammed AfolabiDavid A. EdelmanMutsuyo Takayama-ItoDavide T. MoscaNahed M. Abdel-HaqDelia Mirela TitNatalia SumbatyanDenis Victorovich TikhonenkovNgaio RichardsDenise HarrisonNicole GottdenkerDimitris C. ChatzopoulosNikolaos A. ChrysanthakopoulosDinesh SubediNobuko TunoDolly J. KatzNobumichi KobayashiDominik ŁagowskiNorihiro NishidaDominika GuzekNurudeen Olalekan OlosoDonato TraversaOlga PawełczykDragana M. StanojevicOlga PerovicEdoardo PozioOliver DenisEiji ArakawaPandji Wibawa DhewantaraElisa García-VázquezPaolo ParenteElisabetta RazzuoliParisa GazeraniElivelton Da Silva FonsecaPatricia Silvia RomanoElvina ViennetPau Bosch-NicolauEmilie JavellePaul S. WeissEmilie ValléePaulo BettencourtEmmanouil MagiorkinisPawin PadungtodEric J. NillesPeter ZarbEskild PetersenPetr HorákEto Silas FernandesPhil LoVerdeEzgi AkdesirPier Luigi AcutisF. Matthew KuhlmannPiera MazzoneFabio GomesPilar ForondaFederica FogacciPin-Kuang LaiFerdinando AgrestaPrimrose Beryl GladstoneFrancesca BarattaRachael AubertFrancesca MacchiRaffaele VitielloFrancesco CastelliRavesh SinghFrancesco Di GennaroRegev CohenFranco MutinelliRegina RoweFrank DresslerRemigiusz GałęckiGalya Ivanova GanchevaRhea LongleyGeorge PanosRichard Trevor WilsonGermán KopprioRoberto CalistiGianluca PiatelliRobyn StoddardGillian GileRodolfo CapannaGiuseppe MiserocchiRodrigo Almeida-PaesGouri Rani BanikRoger NasciGrzegorz WoźniakowskiRoger TeckGuoju HongRogério RodriguesH.M. ManukumarRonaldo Cesar Borges GryschekHan Naung TunRose NjeminiHayden HedmanRyan D. CooperHedwin Kitdorlang DkharSaito NobuoHeinz MehlhornSalvatore Giovanni De SimoneHélcio Gil-SantanaSandeep KumarHelene-Mari Van Der WesthuizenSatu HepojokiHenrique PereiraSaurabh RamBihariLal ShrivastavaHeverton DutraSebastian KirchnerHiroshi SatoShailesh KumarHiroyasu SakaiSheikhpour MojganHuibin LvShylo R. JohnsonHuijun ChihSilmara Marques AllegrettiHyun-Ok SongSilvia MascoloIan HodgsonSilvia S. ChiangIan MendenhallSonu SubudhiIbrahim E.A. AbbasSophie HuddartInuk JungStefano CatalanoIoannis A. GiantsisStefano VeraldiIoannis GiantsisSteffen FlessaIosif MarincuStella Maris González CappaIsmael MaatoukStephen GrahamItziar FamiliarStephen MuhiIvana GrgićSteve CarlanJacek ŻmudzkiSteven StoddardJaeson CallaSudhanshu AbhishekJaffar AbbasSulaiman LakohJaindra Nath TripathiSuman MajumdarJames L.L. OcciSusan Beth RifkinJamilah MeghjiTaichiro TakemuraJana KatuchovaTakashi YoshiyamaJanani IyerTerry A. KleinJean PerriotTheresa CoetzerJean VanderpasThiru VanniasinkamJean-Bernard DucheminThomas Robert SheliteJef Van Den EndeThomas ZollerJeffrey BaraTimothy EricksonJennifer D. Foulke-AbelTimothy P. AlgeoJesús NavasTing LiJochen HofstaetterTomas JelinekJohn L. MokiliTomasz BlicharskiJohn McBrideTomislav MeštrovićJohn R. RohdeTomohiro IshikawaJuan C. de la TorreTony Le GallJulián Solís García Del PozoTony SchountzJulien ClaudeToshio HattoriJustyna DunajVeronika Katharina JaegerJyothi F. NagajyothiVictor ArendtKai J. JonasVilla SimoneKaren E. KempsellVincent DelormeKarin LederVishnu RevuriKarolina KotVitrai JózsefKaroun BagamianVladimir SavicKasha Priya SinghWhitney QuallsKatarzyna BartosikWilman CarrilloKathryn Marie JonesXavier VallèsKazuki ShimizuXinzhuan SuKempe Ronald HopeXupeng HongKerry RedicanYalemzewod GelawKonstantinos ArsenopoulosYang HongKovács NóraYueh-Juen HwuKrishna HortZsuzsanna Füzesi

